# Aerobic and Postural Strength Exercise Benefits in People with Schizophrenia

**DOI:** 10.3390/ijerph20043421

**Published:** 2023-02-15

**Authors:** Michele Fonseca Szortyka, Viviane Batista Cristiano, Paulo Belmonte-de-Abreu

**Affiliations:** 1 Graduate Program in Psychiatry and Behavioral Sciences, Federal University of Rio Grande do Sul, Porto Alegre 91501-970, RS, Brazil; 2 Schizophrenia Program, Hospital de Clínicas de Porto Alegre, Porto Alegre 90035-903, RS, Brazil; 3 Schizophrenia Program of the Federal University of Rio Grande do Sul Medical School, Department of Psychiatry, Hospital de Clínicas de Porto Alegre, Porto Alegre 90035-903, RS, Brazil

**Keywords:** functional capacity, physical health, postural exercise, aerobic exercise, schizophrenia

## Abstract

Background: This study aimed to evaluate the effect of two different types of physical intervention on sedentary behavior and clinical changes in people with schizophrenia. Method: This is a clinical trial including people with schizophrenia in regular outpatient care who realized a 3-month exercise protocol and were separated into two groups: aerobic physical intervention (API) and postural physical intervention (PPI). All participants performed an assessment of (a) functional capacity through a 6 min walk test (6MWT), (b) flexibility using Well’s bench, (c) disease severity using the Brief Psychiatric Rating Scale (BPRS), (d) quality of life using the SF-36 Questionnaire and (e) physical activity using the Simple Physical Activity Questionnaire (SIMPAQ). Results: Thirty-eight patients with schizophrenia completed the intervention (24 patients in API and 14 patients in PPI). Regarding sedentary behavior, there was an improvement in the API group in the time exercising and in the PPI group concerning time in bed, time walking and exercising. Regarding quality of life, there was an improvement in the API group (functional capacity) and in the PPI group, there was an improvement in physical limitation, pain and emotional limitations. In the API group, there was an improvement in BMI (body mass index), diastolic blood pressure and systolic blood pressure. Functional capacity was improved only in the PPI group. There was no change in flexibility and disease severity. Conclusions: The study demonstrated a change response in the physical and mental aspects in people with schizophrenia after a change in sedentary behavior.

## 1. Introduction

Schizophrenia is among the most disabling and economically catastrophic medical disorders, ranked by the World Health Organization as one of the top 10 illnesses contributing to the global burden of disease [1]. Symptoms such as hallucinations or delusions; disorganized speech; a flat affect; poverty of speech; and cognitive impairments, including attention, memory and executive functions [2,3], are all associated with social and occupational impairment in these patients.

There is growing interest in the assessment and improvement of global functioning in these patients, with the expansion of physical interventions together with traditional clinical and pharmacological care to increase life expectancy and quality of life. Increased sedentary lifestyles and reduced physical activity are extremely frequent in schizophrenia, which are independent but modifiable risk factors for a decrease in cardiovascular disease and premature mortality in these people [4]. Despite increased mortality and impairment in this population, patients with schizophrenia still have limited access to general health care and a reduced opportunity of illness prevention and treatment compared to the non-psychiatric population [5].

Functional capacity is connected and related to the degree of physical activity within this population [6]. The usual focus has been on the physical health of people with schizophrenia by cardiometabolic risk reduction. Sedentary behavior is extremely prevalent, and about 50% of people with psychosis have reduced physical activity, with only 25% of them fitting the minimum public health recommendation of 150 min of moderate to vigorous physical activity per week [7,8]. Physical inactivity causes a whole body functional impairment, and is strongly associated with negative symptoms and cardiac and metabolic comorbidities (metabolic syndrome and obesity) [9]. An additional systematic review also estimated that patients with schizophrenia are sedentary for about 12 h/day, nearly 3 h more than the control group [10]. 

Regular exercise has positive effects on both the body and mind [11]. Patients with schizophrenia undertaking physical exercise have reduced severity of psychiatric symptoms, decreased positive and negative symptoms and improved quality of life, global functioning and depressive symptoms [12]. Additionally, recent evidence pointed to the impact on brain function and structure in terms of increased neuronal plasticity at the cellular and molecular levels [13,14,15]. Aerobic exercise uses oxygen in the process of generating energy in the muscles, and this type of exercise works a large amount of muscle groups. The evidence of the benefits of aerobic exercise practice in patients with schizophrenia is well documented in several studies [12,15,16]. On the other hand, an exercise that focuses on posture affects body consciousness and motor control. There is a scarcity of studies showing the benefits of postural exercises in patients with mental disorders; however, postural and flexibility interventions play an additional role in the individual’s body representation and behavior, in reducing pain and increasing quality of life [17]. 

The primary aims of the present study were the following: to evaluate the effects of the two techniques—aerobic and postural interventions—over sedentary behavior. The secondary aims were to compare the physical (body mass index, blood pressure, flexibility, functional capacity) and mental (quality of life and disease symptomatology) effects of different interventions in people with schizophrenia.

## 2. Methods

### 2.1. Trial Design

This is a non-randomized clinical trial of two physical interventions (aerobic physical intervention (API) and postural physical intervention (PPI)) over three months in two groups of stable outpatients with diagnosis of schizophrenia. API was applied in patients at a public health facility (Psychosocial Attention Center (CAPS)) in Camaquã, a mid-sized town of southern Brazil, and PPI in patients of the Schizophrenia Outpatient Program (Prodesq) of a major University Hospital—Hospital de Clínicas de Porto Alegre (HCPA)—in a large-sized town in southern Brazil.

### 2.2. Participants

Stable outpatients under regular treatment received a psychiatric diagnosis after a three-step procedure consisting of (1) clinical observation with at least 3 evaluations; (2) a family interview; and (3) a review of their medical records performed by a trained psychiatrist. The selected patients met the following inclusion criteria: Diagnostic and Statistical Manual of Mental Disorders (DSM-5) [18] and International Classification of Diseases (ICD)-10 diagnoses of schizophrenia [19]; were aged between 18 and 65 years; were under stable drug treatment adjusted to their clinical state for at least 3 months; and were not involved in other physical activity programs during the intervention. Exclusion criteria were alcohol or other drug abuse in the last month; major systemic or neurological diseases; physical disability contraindicating physical activity; risk of suicide confirmed by direct contact with the patient and family; pregnancy or women of reproductive age that did not use a contraception method; and not agreeing to participate in the study after full explanation of the program. 

### 2.3. Ethics

The study was registered in the Brazilian Research Registry under No. 43408615.7.0000.5327, in the Brazilian Registry of Clinical Trials (ReBEC) under No. RBR-2h2hjy and approved (150066) by the Research Ethics Committee of Hospital de Clínicas de Porto Alegre (HCPA). Patients and their legal guardians provided written informed consent after reading and understanding the intervention program and their rights.

### 2.4. Clinical Assessment

After patient recruitment, previously trained professionals performed a standardized clinical and physical assessment of the study to participants before physical intervention and after 3 months of treatment.

### 2.5. Sedentary Lifestyle

Measured by the Simple Physical Activity Questionnaire (SIMPAQ). It is a 5-item clinical tool designed to assess physical activity among populations at high risk of sedentary behavior. The physical activity questionnaire evaluates the last seven days and includes time in bed, sedentary time, time spent walking, type and time spent exercising, and time spent in other activities, including leisure, domestic, work and transportation activities [20].

### 2.6. Disease Severity

Measured by the Brief Psychiatric Rating Scale (BPRS). It is one of the most widely used instruments to evaluate the presence and severity of various psychiatric symptoms including the Brazilian Unified Health System (SUS) for patient’s monitoring [21]. It assesses 18 domains of symptoms: worry about body; anxiety; withdrawal; conceptual disorganization; guilt; tension; mannerism; grandiosity; depressive mood; hostility; paranoid ideation; hallucination; psychomotor retardation; lack of cooperation; delusions; affect; excitement and disorientation. The assessment takes approximately 5–10 min, following an interview with the patient, and the clinician rates each item on a scale ranging from 0 (not present) to 6 (extremely severe) through observation and questions depending on the assessed item. 

### 2.7. Physical Performance

Measured by the Six-Minute Walking Test (6MWT). Two trained and certified physical therapists applied 6MWT following the American Thoracic Society Guidelines (2002) [22], in a corridor with demarcated turnaround points with minimal external stimuli, with participants walking as briskly as possible, without running, for 6 min. They should do their best during this time, stopping if needed, and technicians would use standard encouraging words and attitudes throughout the test. Researchers monitored blood pressure, heart rate, respiratory rate, peripheral blood oxygen saturation and dyspnea (measured by Borg’s perceived exertion scale) at the first, third and sixth minute. Algorithms by Enright and Sherrill (2003) [23] predicted ranges for 6 min walking distances (6MWD) using sex, height, age and weight parameters.

### 2.8. Stretching

Measured by the Well’s Bench sit and reach flexibility test. It measures lower limb and hip joint flexibility, with the participant fully extended the legs, with the soles of their feet against the bench, slowly bending and projecting forward as far as possible, and fingers sliding along a scale. The total distance reached after three (3) attempts provided the final score [24]. 

### 2.9. Quality of Life

Measured by the Medical Outcomes Study 36-Item Short Form—SF-36. This instrument is a validated questionnaire method with high sensitivity to detect functional status, among other aspects of quality of life. It includes eight multiple-item subscales of functional capacity, physical limitation, pain, general health, vitality, social aspects, emotional limitations and mental health. The total score on each SF-36 subscale ranges between 0 and 100, and higher the score is, the better the patient is [25]. 

### 2.10. Physical Intervention

#### 2.10.1. Aerobic Physical Intervention [API]

This 12-week program included a 1 h session of aerobic exercise twice a week. It measures exercises by a digital rate monitor (POLAR FT1^®^, https://support.polar.com/e_manuals/FT1_FT2/Polar_FT1_FT2_user_manual_English/manual.pdf, accessed on 22 August 2022) with adjustment by age, sex, weight and height. Measurements ranged from 70% to 80% of maximum heart rates calculated by the Karvonen formula [26]. The session began with a 5 min warm-up of comfortable intensity and continued with an aerobic exercise of increasing intensity with any of the 3 modalities: 1. stationary bicycle (Embreex 367C, Brusque, Brazil), 2. Treadmill (Embreex 566BX, Brazil) or 3. Elliptical trainer (Embreex 219, Brazil). This strategy followed public health recommendations for adaptation to individual preferences in schizophrenia [9,27]. A trained professional provided guidance, equipment adjustment and participant’s encouragement of the exercise performance. After aerobic exercise, participants performed large muscle stretching. Heart monitors recorded initial and maximum heart rate and calories expended during a session.

#### 2.10.2. Physical Intervention

Postural Physical Intervention [PPI]: The 12-week program included twice a week 1 h sessions of postural exercises. Trios or quartets of subjects received an intervention, beginning with a 5 min warm-up with a stationary walk, followed by 15 min of muscle flexibility and joint mobility exercises. After 25 min of global muscle resistance exercises (the muscular groups emphasized were the paravertebrals, abs, extenders, flexors, adductors, hip abductors, shoulders, knees and elbows flexors and extenders), the session ended with 15 min of body awareness work through breathing. The evolution of the exercises, as well as the use of accessories such as balls, elastics, dumbbells, etc., respected the individuality of each one. A properly trained professional conducted the consultations, demonstrating the exercises and correcting them when necessary.

### 2.11. Statistical Analyses 

Statistical analyses used SPSS version 20.0, with categorical variables described by frequencies and percentages, and tested for symmetry by the Kolmogorov Smirnov test. Quantitative variables with symmetrical distribution were expressed by means and standard error or means and standard deviation, and those with asymmetric distribution by median, minimum and maximum. Quantitative variables with symmetrical distribution used a Student’s *t* test. A Generalized Estimating Equations (GEE) model was performed to analyze the variation over time of the variables and the between group interaction over time. This method analyzes the data using an intention-to-treat approach. The significance level was 5%.

## 3. Results

**Figure 1:**
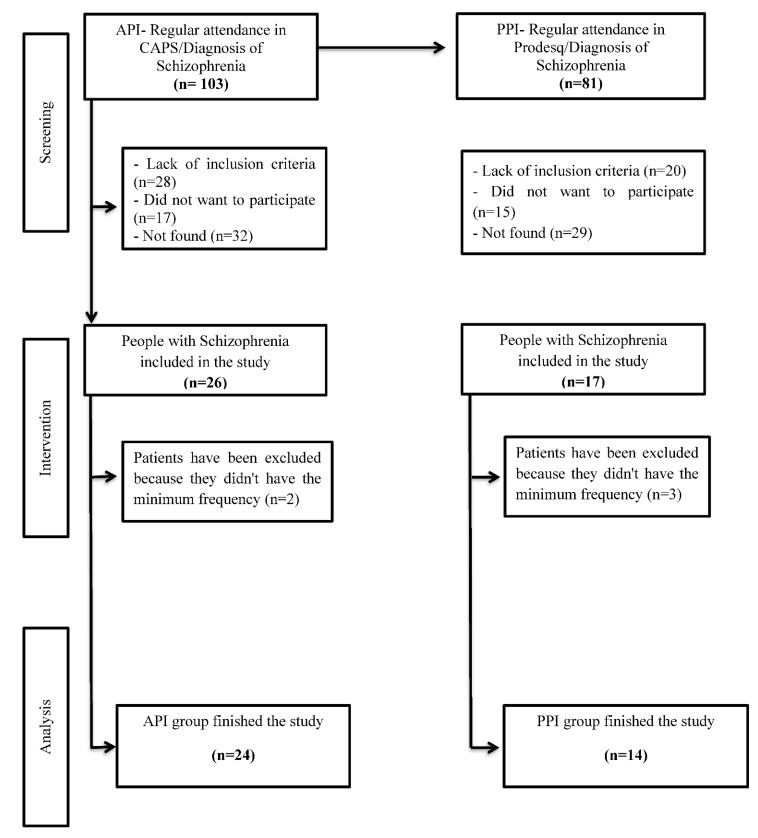
Study Flowchart: Aerobic physical intervention (API) and postural physical intervention (PPI) in people with schizophrenia.

Out of the 103 patients in the API group that were initially invited to participate, 26 agreed to participate and met the inclusion criteria. Of these, 24 patients (92%) completed the physical exercise intervention. Dropouts consisted of two patients (8%) that did not achieve the minimum attendance. Regarding in PPI group derived from 81 patients that received invitation to the study, from these, 17 agreed to participate. Of these, 14 patients (82%) completed the postural intervention. Dropouts consisted of three patients (18%) that did not achieve minimum attendance (Figure 1).

The two groups were homogeneous, except in the body mass index (BMI). The API group had higher BMI (*p* = 0.014) and the analyses were adjusted for BMI. The mean weight in the API group was reduced from 89.4 kg (SE = 5.00) to 87.4 kg (SE = 4.88) after intervention (*p* = 0.005); the same decreasing effect was observed in body mass index (BMI) (*p* < 0.001). The mean weight of participants in this PPI group was 74.1 kg (SE = 5.07) and after the PPI it was 74.7 kg (SE = 5.08) (*p* = 0.078), and BMI decreased (*p* = 0.038). In the API group, the mean flexibility increased from 16.4 cm (SE = 1.71) to 17.4 cm (SE = 1.76) after intervention (*p* = 0.299) and the PPI mean flexibility was not significant (pre 18.8 cm (SE = 1.98) to 19.3 cm (SE = 1.89) after intervention (*p* = 0.173)). Data is shown in Table 1. 

In the API group, the systolic blood pressure decreased from 125 mmHg (SD = 10.6) to 121.3 mmHg (SD = 11.2) and diastolic blood pressure decreased from 82.9 mmHg (SD = 10.8) to 77.9 mmHg (SD = 11.8), (*p* < 0.001 and *p* = 0.001, respectively). In the PPI group, systolic blood pressure remained the same after the intervention 114.3 (SD = 8.5) and diastolic blood pressure increased from 75.7 mmHg (SD = 9.4) to 79.3 mmHg (SD = 9.2), (*p* = 0.026 and *p* = 0.100, respectively). The interaction between groups was systolic blood pressure (*p* = 0.129) and diastolic blood pressure (*p* = 0.001).

On the Simpaq scale (Table 2), the API group showed significant improvement in exercising domain (*p* < 0.001) and the PPI group improved in time in bed (*p* = 0.040), time walking (*p* < 0.001) and exercising (*p* < 0.001). The interaction between groups was in time walking (*p* = 0.051) and exercising (*p* = 0.032). 

Regarding quality of life, as quantified by the questionnaire SF-36 (Table 2), there was an improvement in functional capacity in the API group (*p* = 0.004). The PPI group showed an improvement in physical limitation (*p* = 0.016); pain (*p* = 0.006) and emotional limitations (*p* = 0.033). The difference between the groups was only in pain domain (*p* = 0.013).

**Figure 2:**
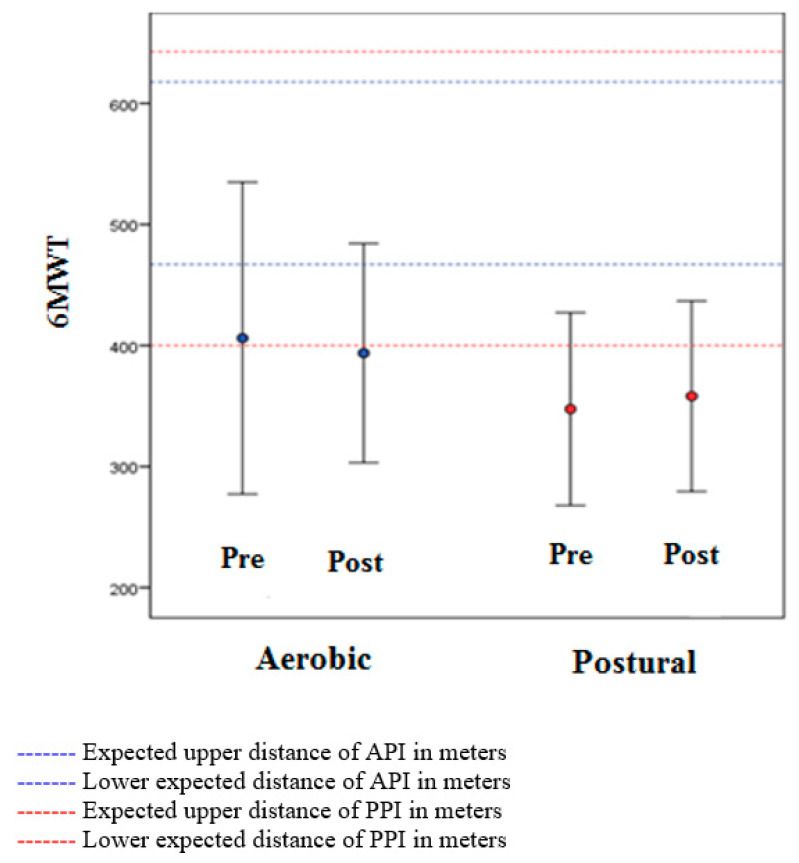
Relationship between the distances walked in the 6 min walking test (6MWT) and the upper and lower limit predicted of patients who performed the aerobic physical intervention (API) and 14 cases that performed the postural physical intervention (PPI) in people of schizophrenia.

Regarding physical performance, 6MWT (Figure 2), patients in the API group initially walked 406 (SD = 128) meters and after the intervention 391 (SD = 90) meters (*p* = 0.588). The PPI group initially walked 347 (SD = 79) meters and 358 (SD = 78) meters (*p* = 0.003) after the intervention. There was no significant difference between groups (*p* = 0.269).

BPRS changes were similar among the API and PPI groups. There was no difference in all 18 items between groups, i.e., (1) Worry about body (*p* = 0.393); (2) Anxiety (*p* = 0.201); (3) Withdrawal (*p* = 0.870); (4) Conceptual disorganization (*p* = 0.212); (5) Guilt (*p* = 0.463); (6) Tension (*p* = 0.856); (7) Mannerism (*p* = 0.988); (8) Grandiosity (*p* = 0.846); (9) Depressive mood (*p* = 0.643); (10) Hostility (*p* = 0.777); (11) Paranoid ideation (*p* = 0.940); (12) Hallucination (*p* = 0.988); (13) Psychomotor retardation (*p* = 0.964); (14) Lack of cooperation (*p* = 0.622); (15) Delusions (*p* = 0.687); (16) Affect (*p* = 0.482); (17) Excitement (*p* = 0.687) and (18) Disorientation (*p* = 0.601).

## 4. Discussion

The major finding of the study was that the aerobic physical intervention program reduced weight, body mass index and blood pressure and increased time exercising, whereas the postural physical intervention showed an improvement in sedentary behavior through SIMPAQ and 6MWT. No effects were seen in flexibility and disease severity in both groups.

The increase in the practice of physical activity and the reduction of sedentary behavior are gaining more visibility in research and clinical attention due to the feasibility and effectiveness of exercise programs integrated into the range of therapeutic approaches to schizophrenia. On the SIMPAQ scale in the initial assessment, the PPI group spent more time staying in bed than the API group; the API group initially spent 41%/day sleeping and after the intervention spent 40%, and the PPI group spent 45%/day sleeping and after the intervention spent 42%. People with schizophrenia engage in less physical activity than the general population [10]. In a study where it was possible to quantify the level of physical activity in 3453 patients with schizophrenia, there was an average of 80 min of light exercise, 47 min of moderate to vigorous exercise and 1 min of vigorous exercise per day (well below the recommended values) [28].

Patients in both groups received no specific dietetic orientation, and only API had an effect on BMI (*p* = 0.002). This indicates a positive effect on obesity and cardiovascular health in this group, complicated by the fact that people diagnosed with schizophrenia tend to have about two times more obesity and cardiovascular problems compared to the general population. Physical inactivity is associated with cognitive and negative symptoms, as well as with increased BMI. Most of the weight gain was caused by antipsychotic medications [7]. Weight loss is a major challenge for patients with schizophrenia. Recent findings on the effectiveness of exercise interventions to address obesity are ambiguous. Aerobic exercise reduced BMI in schizophrenia patients [29], as did soccer practice [30], but the meta-analysis by Vancampfort [31] reported that exercise improved cardiorespiratory fitness without a BMI reduction. In the PPI group, there was no decrease in weight as expected because of the low energy expenditure that exercise requires.

The study evidenced a significant improvement in several domains of the SF-36 scale, in support of several previous studies demonstrating the need for new interventions to increase the quality of life in patients diagnosed with schizophrenia. The PPI group showed similar effects of other interventions such as yoga and dance. Cramer [32] found that yoga had moderated short-term quality of life improvement in patients with schizophrenia, and Kaltsatou [33] implemented an 8-month dance program with significant improvement in psychopathology and quality of life compared to controls without a dance program. Dauwan [12] pointed to the additional effect of combining aerobic exercise with yoga.

Several physiological effects of stretching included elastic property referring to the capability of the tendon muscle unit to return to its original length after being stretched; the analgesic effect; the anti-inflammatory effect in the injuries of muscle fibers, among others [34]. In the present study, patients failed to achieve improvement by stretching, but this information may be useful in future studies, since the literature of stretching in people with mental disorders is very scarce. The closest we came to reaching a patient with mental disorders were patients with fibromyalgia, whereby the stretching exercises improved the quality of life (measured by the SF-36 scale) [35], with a more pronounced effect on physical functioning and pain, and where resistance training was more effective in reducing depression. Additionally, an aquatic protocol using stretching exercises in depressed elderly people was associated with a 44% improvement in flexibility [36].

On the other hand, unexpectedly, the functional capacity in the API group did not show significant improvement after the intervention, only in the PPI group. Patients in the API group were 13% below the predicted minimum level and after the intervention remained as such (15% below the expected) and patients in the PPI group were 29% below the minimum predicted level, improving 27%. This also occurred in other studies. Vancampfort [37,38] showed that overweight patients walked a shorter mean distance in the 6MWT than patients who are not overweight, and reported that this could derive from the side effects of antipsychotics and musculoskeletal pain. 

The limited effect of interventions can relate to the following study limitations: (1) limited sample size that may have interfered with our result; and (2) limited time intervention (perhaps the 3 month duration in physical interventions did not provide enough time for clinical and functional response). The worsening of some aspects such as the total BPRS scores or the unexpected results of the 6MWT represents a great challenge. We should have included a third group of patients with schizophrenia who did not realize any physical intervention. In this article, we do not consider the doses and types of antipsychotic drugs or the side effects that they can cause; however, they should be considered in future studies. Another important limitation is the difficulty to standardize specific exercise, real effort dispended and type, frequency and intensity of practice. These factors can affect the outcomes. Additionally, we must consider the effect of the difficulty in patient management and patient motivation affecting observed outcomes.

## 5. Conclusions

In conclusion, the aim of the present article was to provide a modification of sedentary behavior through aerobic and postural exercise in people with schizophrenia, in addition to observing its effects on physical and mental health. This study showed that both protocols were effective in this population, which are otherwise lacking in complementary therapies. The aerobic physical intervention improved physical health outcomes such as weight, BMI, blood pressure and more time exercising. The postural physical intervention improved the sedentary behavior (SIMPAQ and 6MWT) and quality of life. However, clinical studies are still needed to develop better evidence-based physical training programs for patients with schizophrenia, including greater accessibility and better protocols for types and intensities of exercises.

## Figures and Tables

**Table 1:** Table of sample characteristics of 24 cases that performed the aerobic physical intervention (API) and 14 cases that performed the postural physical intervention (PPI) in people with schizophrenia.

Variables	Aerobic N = 24 (63.2%)	Postural N = 14 (36.8%)	*p* *
Age, mean ± SE	39.3 ± 2.55	43.78 ± 2.33	0.245
Gender Male, n (%)	20 (83.3)	12 (85.7)	0.999
Basic education, n (%)	24 (100)	14(100)	1.000
Single marital status, n (%)	23 (95.8)	14(100)	0.999
Smoker, n (%)	7 (29.2)	7(50)	0.298
Height pre, mean ± SE	1.68 ± 0.01	1.70 ± 0.27	0.378
Weight pre, mean ± SE	89.4 ± 4.99	74.13 ± 5.07	0.053
BMI pre, mean ± SE	31.6 ± 1.72	25.15 ± 1.36	0.014
Chronicity > 7 years, n (%)	20 (83.3)	14 (100)	0.276
Hospitalization (median, minimum and maximum)	2 (0 and 20)	1 (0 and 5)	0.120
Flexibility pre, mean ± SE	16.4 ± 1.71	18 ± 3.38	0.644

SE, Standard error; * Student’s *t* test, *p* < 0.05; BMI, body mass index.

**Table 2:** Table with the pre- and post-SF-36 quality of life scale and SIMPAQ scale of the 24 cases who realized aerobic physical intervention (API) and 14 cases who realized postural physical intervention (PPI) in people with schizophrenia.

	Aerobic N = 24		Postural N = 14		*p* * API	*p* * PPI	*p* * Interaction
SF-36	Pre	Post	Pre	Post			
Functional capacity	72.50 (0–100)	85 (40–100)	77.50 (0–95)	75 (30–100)	0.004	0.630	0.187
Physical limitation	37.50 (0–100)	50 (0–100)	12.50 (0–100)	75 (0–100)	0.455	0.016	0.211
Pain	72.00 (20–100)	71 (0–100)	55.50 (11–100)	82 (50–100)	0.520	0.006	0.013
General health	46.00 (10–87)	54.50 (15–100)	54.50 (30–87)	65 (15–92)	0.209	0.502	0.819
Vitality	60.00 (0–95)	65 (15–100)	60.00 (20–85)	62.50 (10–100)	0.354	0.485	0.825
Social aspects	50.00 (0–100)	68.50 (37–100)	63.75 (0–100)	75 (0–100)	0.132	0.315	0.952
Emotional limitations	16.50 (0–100)	83 (0–100)	0.00 (0–100)	100 (0–100)	0.063	0.033	0.638
Mental health	76.00 (0–100)	68 (8–100)	62.00 (0–100)	74 (28–96)	0.778	0.566	0.728
SIMPAQ							
Time in bed	600 (420–840)	570 (420–750)	660 (330–945)	600 (520–840)	0.467	0.040	0.246
Sedentary time	375 (60–660)	2 (120–780)	155 (0–780)	210 (0–540)	0.905	0.151	0.486
Time walking	75 (0–840)	115 (0–1260)	55 (0–780)	120 (0–800)	0.383	< 0.001	0.051
Exercising	0 (0–180)	80 (80–680)	0 (0–0)	120 (0–210)	< 0.001	< 0.001	0.032
Other Activities	0 (0–360)	0 (0–300)	0 (0–60)	0 (0–90)	0.809	0.237	0.781

Data is presented as median (minimum–maximum); * *p*-Value: Analyzed by GEE (Generalized Estimating Equation Models); *p* < 0.05. SF-36 (quality of life scale); SIMPAQ (simple physical activity questionnaire); API (aerobic physical intervention); PPI (postural physical intervention).
